# Transient Whitish Change in the Gastric Mucosa Induced by Water‐Jet Flushing

**DOI:** 10.1002/deo2.70448

**Published:** 2026-10-06

**Authors:** Hidetaka Hamamoto, Hiroaki Saito, Tomoki Matsuda

**Affiliations:** ^1^ Department of Gastroenterology Caress Memorial Hospital Hokkaido Japan; ^2^ Department of Gastroenterology Sendai Kosei Hospital Miyagi Japan; ^3^ Department of Gastroenterology Soma Central Hospital Fukushima Japan

**Keywords:** endoscopic mucosal resection, gastric mucosa, gastroscopes, pallor, therapeutic irrigation

## Abstract

Localized transient whitening of the gastric mucosa after water‐jet flushing through the auxiliary water channel is rarely described. We report a 49‐year‐old man who underwent endoscopic submucosal dissection for a 5‐mm gastric‐type neoplasm on the greater curvature of the lower gastric body. During washing of adjacent non‐lesional mucosa, strong auxiliary water‐channel flushing for approximately 2–5 s was followed by a focal whitish linear change confined to the flushed area, which became evident approximately 10 s after flushing was stopped and persisted for approximately 30 s before resolving spontaneously, without symptoms or adverse events. Similar changes were observed on the following day and 84 days after resection, but not under the recorded conditions of weaker auxiliary water‐channel flushing or manual instrument‐channel spraying. When a localized whitish change is observed after strong auxiliary water‐channel flushing, brief observation for spontaneous resolution may help distinguish this transient finding from a fixed mucosal lesion.

**Trial Registration**: N/A

## 1 Introduction

Whitish changes in the gastric mucosa have been described in chronic ischemic gastritis, peristaltic blanching in chronic mesenteric ischemia, and reversible ischemic‐appearing change induced by gastric wall expansion during air insufflation [1, 2, 3]. Here, we describe focal, short‐lived whitish changes observed after strong water‐jet flushing through the auxiliary water channel (hereafter, auxiliary water‐channel flushing) before and after endoscopic submucosal dissection (ESD).

## 2 Case Report

A 49‐year‐old man was referred for endoscopic treatment of a 5‐mm lesion on the greater curvature of the lower gastric body diagnosed as a gastric‐type adenoma (Figure 1a). He had no vascular risk factors or medications. Serum anti‐*Helicobacter pylori* antibody was <3 U/mL, the stool *H. pylori* antigen test was negative, endoscopy showed no antral atrophy, and there was no history of eradication therapy, supporting *H. pylori*‐uninfected status. A GIF‐XZ1200 endoscope and OFP‐2 flushing pump (Olympus, Tokyo, Japan) were used. Additive‐free degassed water at room temperature was used for auxiliary water‐channel flushing throughout; a separately prepared dimethicone‐containing tap‐water solution was used only for manual instrument‐channel spraying. On the day of ESD, during washing of the mucosa adjacent to the lesion, auxiliary water‐channel flushing was performed for approximately 2–5 s at output setting 8 or 9 on the nine‐level indicator (our routine washing range). The jet was swept along the mucosa rather than held at a single point, such that each point was struck only briefly. This was followed by a focal whitish linear change confined to the path of the jet on the greater curvature of the lower gastric body and, after further flushing, a similar change at the anterior wall side of the greater curvature of the middle gastric body (Figure 1b). On video review (Video S1), each change became evident approximately 10 s after flushing was stopped and persisted for approximately 30 s before resolving spontaneously (Figure 1c). The change tended to occur when the outlet was relatively close to the mucosa, and the jet struck it nearly perpendicularly. It also occurred at greater distances when nearly perpendicular, whereas no clear change was observed in the single recorded instance of oblique impact. Distance and angle were neither measured nor standardized. No symptoms or adverse events occurred, and ESD was then performed.

**FIGURE 1:**
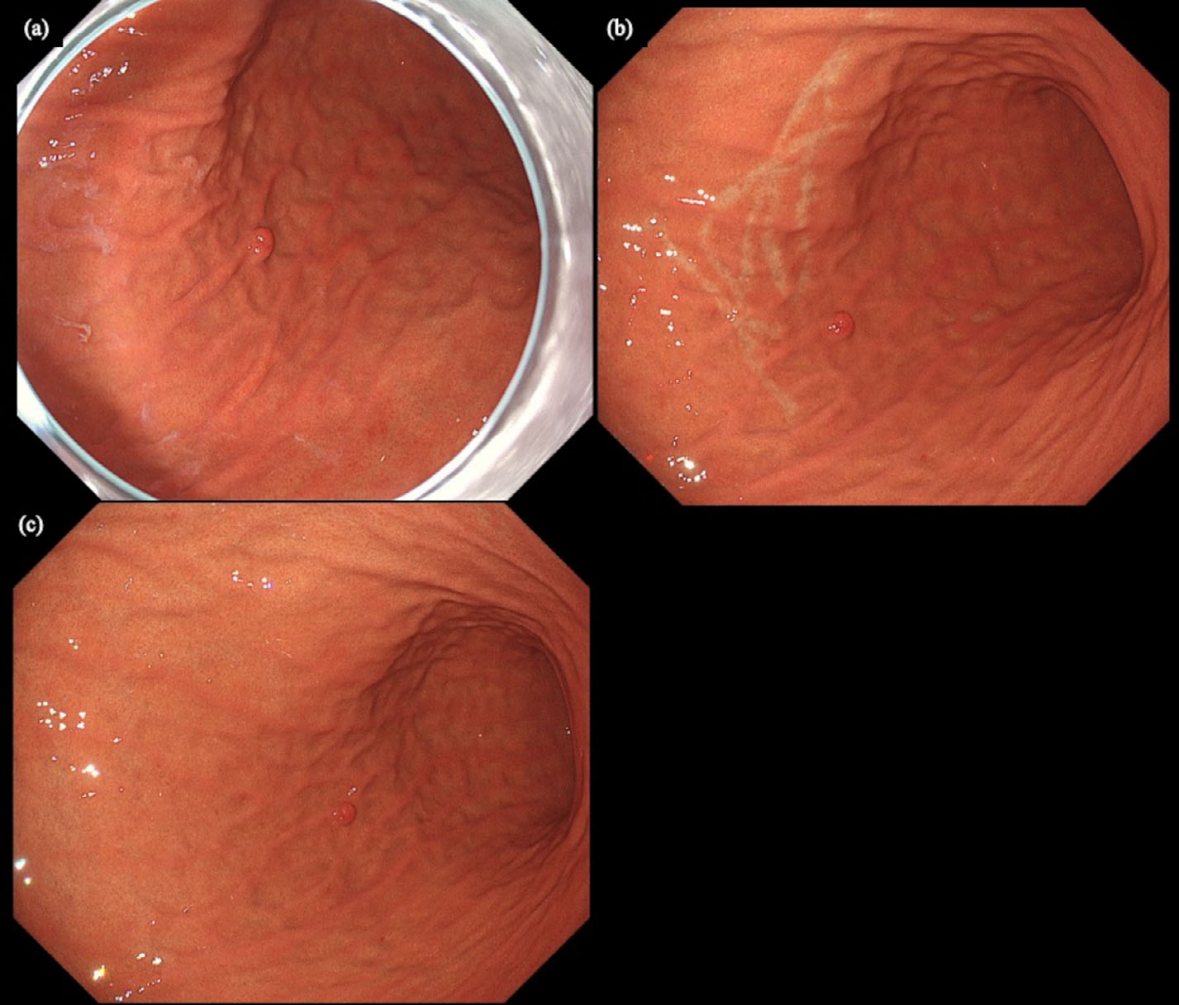
Endoscopic findings on the day of endoscopic submucosal dissection (ESD), shown on white‐light imaging. (a) A lesion on the greater curvature of the lower gastric body that was initially diagnosed as a gastric‐type adenoma. (b) A focal whitish linear mucosal change at the anterior wall side of the greater curvature of the middle gastric body became evident approximately 10 s after strong auxiliary water‐channel flushing (approximately 2–5 s) was stopped; this was the second of two changes observed on this day, the first having appeared at the greater curvature of the lower gastric body adjacent to the lesion (shown in Video S1). (c) The change persisted for approximately 30 s and then disappeared spontaneously.

Histopathology showed a completely resected, well‐differentiated adenocarcinoma of the gastric type. The two whitish changes were located slightly oral and anal to the lesion; the specimen lay between them and included neither site. In the non‐neoplastic mucosa and submucosa adjacent to the tumor, no evident epithelial injury, lamina propria edema, vascular dilatation, or thrombosis was identified (Figure 2a,b). However, these findings do not represent the whitish changes. Contrast‐enhanced computed tomography showed no major vascular stenosis or calcification. On the following day, during endoscopy for postoperative assessment, routine washing of the anterior wall of the middle gastric body adjacent to the post‐ESD ulcer at output setting 8 or 9 was followed by a similar change with the same time course (Figure 2c). No further flushing was performed, and the change resolved spontaneously. Moreover, it was observed on texture and color enhancement imaging mode 1 (Figure 2d).

**FIGURE 2:**
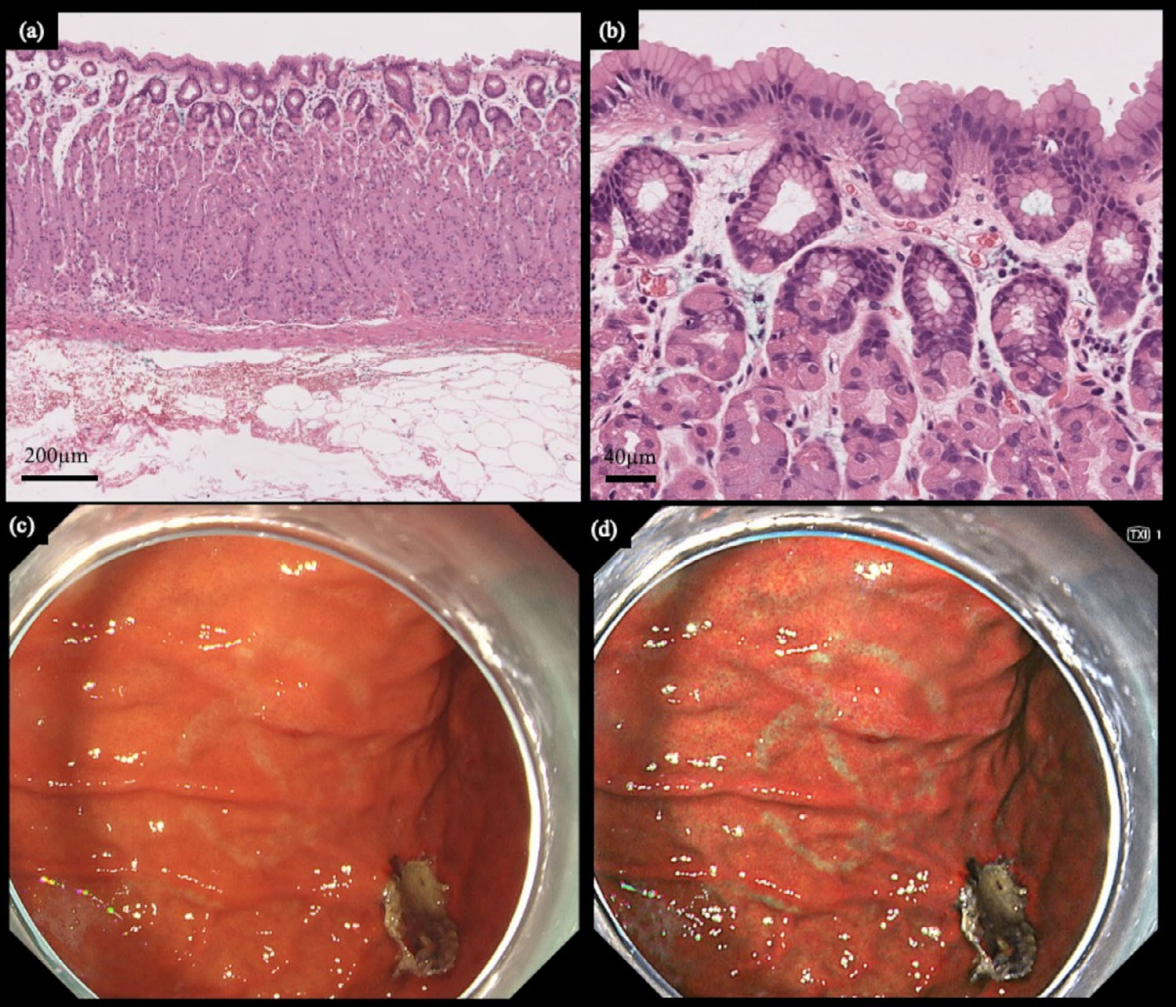
Histopathological findings of the endoscopic submucosal dissection (ESD) specimen (a, b) and endoscopic findings on the day after ESD (c, d). (a, b) Low‐power (a) and high‐power (b) images of non‐neoplastic mucosa and submucosa adjacent to the lesion (hematoxylin and eosin; scale bars as indicated), showing no evident epithelial injury, lamina propria edema, vascular dilatation, or thrombosis. The whitish sites were not included in the specimen. (c) On the day after ESD, a similar focal whitish linear mucosal change was observed at the anterior wall of the middle gastric body, adjacent to the post‐ESD ulcer, after strong auxiliary water‐channel flushing (white‐light imaging). (d) The same site on the day after ESD on texture and color enhancement imaging (TXI) mode 1.

Routine surveillance endoscopy 84 days after ESD showed complete ulcer healing without residual neoplasia (Figure 3a). After manual instrument‐channel spraying, routine washing of the anterior wall of the middle gastric body at output setting 8 or 9 was followed by a focal whitish linear change with the same time course (Figure 3b,c). No further flushing was applied until it had resolved.

**FIGURE 3:**
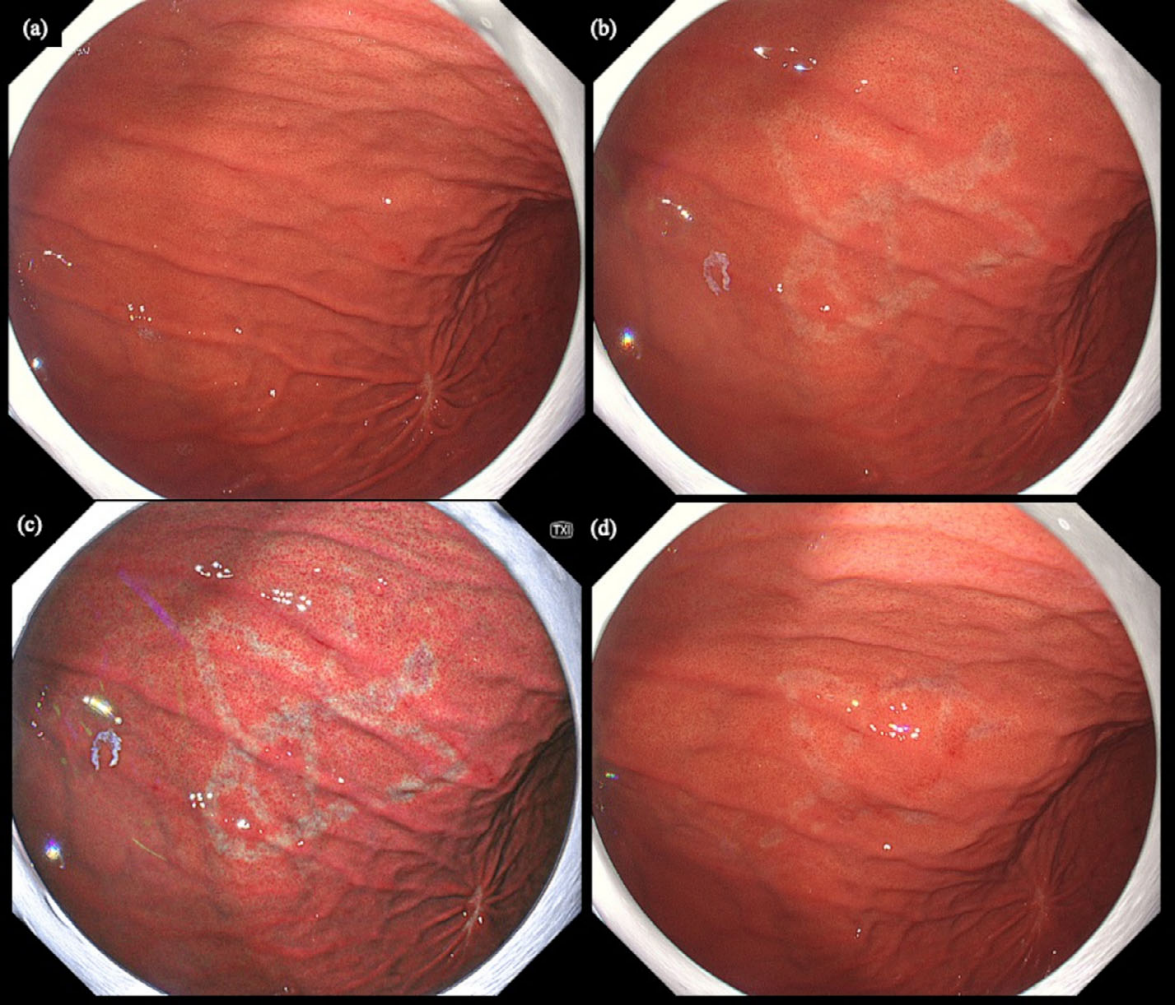
Endoscopic findings at surveillance endoscopy 84 days after endoscopic submucosal dissection (ESD). (a) ESD scar on the greater curvature of the lower gastric body (white‐light imaging). (b, c) The first of two similar focal, whitish linear changes observed during this examination on the anterior wall of the middle gastric body, approximately 10 s after strong auxiliary water‐channel flushing was stopped, shown on white‐light imaging (b) and texture and color enhancement imaging (TXI) mode 1 (c); this episode was documented by still images only. (d) The second change at the same site, which appeared after instrument‐channel spraying and flushing at output setting 7 or lower had produced no change, and the same brief flushing was repeated after complete resolution of the first change (white‐light imaging); this episode is shown in Video S1.

After complete resolution, weaker delivery was tested by manually injecting 20 mL of the dimethicone‐containing solution over approximately 1.7 s through the 2.8‐mm instrument channel using a 30‐mL syringe, without a pump or accessory (approximately 706 mL/min calculated; velocity and pressure not measured), followed by auxiliary water‐channel flushing at output setting 7 or lower at the same site; no marked change was observed. Routine washing at output setting 8 or 9 with a nearly perpendicular jet was then resumed once, and a second similar transient change appeared at the same site and resolved spontaneously (Figure 3d; Video S1).

Similar transient changes were observed twice on the day of ESD, once on the following day, and twice 84 days after ESD, across three clinically indicated examinations. These observations were not protocol‐based, included no systematic comparison among sites, and do not establish reproducibility or flow‐rate dependence.

## 3 Discussion

The principal finding was a focal, short‐lived, jet‐conforming whitish change observed after strong auxiliary water‐channel flushing during three examinations, without persistent ischemic change. To our knowledge, a localized linear whitish change conforming to an additive‐free water jet delivered through the auxiliary water channel and resolving shortly after flushing stopped has not been reported. Ischemic gastritis and mesenteric ischemia‐related blanching are typically patchy or geographic and related to splanchnic hypoperfusion or peristalsis [1, 2], and insufflation‐related change reflects wall expansion [3]. In contrast, the present change was confined to the flushed site, appeared only after flushing stopped, and resolved within approximately 30 s, suggesting association with localized water‐jet exposure rather than fixed ischemia.

The mechanism remains unproven because mucosal blood flow was not measured. We consider transient reduction of superficial mucosal microcirculation from brief mechanical compression by the jet the hypothesis most consistent with the jet‐conforming linear morphology, the tendency to occur with a close, nearly perpendicular jet, and spontaneous resolution after flushing stopped. Increased intraluminal pressure reduces gastric mucosal blood flow on laser‐Doppler flowmetry [4], although demonstrated with sustained air distension rather than a localized jet. Differences in gastric distension or wall tension may also have influenced the change [3, 4]. Normal contrast‐enhanced computed tomography does not exclude a localized, transient reduction in microvascular perfusion. As biopsy during the whitening, magnifying narrow‐band imaging before, during, and after it, and measurement of mucosal perfusion during it were not performed, an optical effect from disturbance of the superficial mucus or water layer cannot be excluded.

In a subsequent bench measurement (another unit of the same endoscope and pump models, with different tubing and pump head), auxiliary water‐channel output into air was approximately 170, 177, and 187 mL/min at settings 7, 8, and 9 (three 20‐s collections per setting). The change was observed at settings 8 and 9 but not at 7. However, because output differed only slightly among these settings and distance, angle, and mucosal pressure were not controlled, it cannot be determined whether this apparent threshold reflects volumetric flow, jet velocity or pressure, or other factors. Although the calculated delivery rate of the manual instrument‐channel bolus exceeded these outputs, no whitening was observed. We therefore hypothesize that the narrower auxiliary water‐channel outlet may have produced a faster jet and greater local pressure on the mucosa and that these factors, rather than the volume delivered, contributed to the change. This interpretation remains speculative because the delivery pattern, fluid, distance, and angle differed or were not standardized.

No misdiagnosis, additional biopsy, or unnecessary intervention occurred, and its clinical relevance is limited. Here, the linear shape conforming to the moving jet was readily recognizable. However, because the whitening reflected the flushed area, a stationary or broadly applied jet might produce a patchy change resembling a flat whitish lesion. If its relationship to flushing is overlooked, such an area could prompt further evaluation or unnecessary searching. Spontaneous disappearance within approximately 30 s, without any change in insufflation or other intervention [3], may help distinguish it from a fixed neoplastic lesion and from ischemic [1, 2] or chemically induced injury, which is generally more persistent and may show erosion, bleeding, or edema. When a localized whitish change is observed after strong flushing, brief observation for spontaneous resolution before additional intervention may therefore be useful.

This report is limited by single‐patient, non‐protocol‐based observations and the absence of systematic comparisons among gastric sites; the frequency, patient or site specificity, reproducibility under standardized conditions, and generalizability remain uncertain. No patient characteristic explaining the finding was identified. Flow rate and mucosal pressure were not measured during the examinations; the pump‐head condition was not recorded, and jet distance and angle, gastric distension and wall tension, and fluid temperature were not standardized. The mechanism remains speculative, and the ESD specimen did not include the whitish sites.

## Author Contributions

**Hidetaka Hamamoto**: conceptualization, data curation, investigation, visualization, project administration, and writing – original draft. **Hiroaki Saito** and **Tomoki Matsuda**: supervision and writing – review & editing.

## Funding

The authors have nothing to report.

## Ethics Statement

This study was conducted in accordance with the Declaration of Helsinki.

## Consent

The patient consented to the clinical examinations, and written informed consent was obtained from the patient for publication of the case details, endoscopic images, and videos.

## Conflicts of Interest

The authors declare no conflicts of interest.

## Supporting information




**VIDEO S1** Transient whitish linear changes observed after auxiliary water‐channel flushing during three clinically indicated endoscopic examinations: on the day of ESD, the day after ESD, and 84 days after ESD. For each examination, the video shows brief auxiliary water‐channel flushing at OFP‐2 output setting 8 or 9 for approximately 2–5 s, followed by the appearance of a focal whitish linear change approximately 10 s after flushing was stopped and spontaneous resolution within approximately 30 s. On the day of ESD, both changes are shown: the first on the greater curvature of the lower gastric body adjacent to the lesion and the second on the anterior wall side of the greater curvature of the middle gastric body. On the following day, during endoscopy for postoperative assessment, routine washing of the anterior wall of the middle gastric body adjacent to the post‐ESD ulcer at output setting 8 or 9 was followed by a similar change with the same time course. For the examination 84 days after ESD, the video shows the sequence after complete resolution of the first change (documented in Figure 3b,c): instrument‐channel spraying and flushing at output setting 7 or lower at the same site, after which no marked mucosal change was observed, followed by resumption of the usual washing at output setting 8 or 9 and the appearance of a second similar change (Figure 3d), which again resolved spontaneously.

## Data Availability

The data that support the findings of this study are available on request from the corresponding author. The data are not publicly available due to privacy or ethical restrictions.
